# Research on the Jumping Control Methods of a Quadruped Robot That Imitates Animals

**DOI:** 10.3390/biomimetics8010036

**Published:** 2023-01-15

**Authors:** Kang Wang, Haoyu Zhao, Fei Meng, Xiuli Zhang

**Affiliations:** 1 School of Mechanical, Electronic and Control Engineering, Beijing Jiaotong University, Beijing 100044, China; 2 Intelligent Robotics Institute, School of Mechatronical Engineering, Beijing Institute of Technology, Beijing 100081, China

**Keywords:** quadruped robot, jumping motions, trajectory optimization, model predictive control

## Abstract

At present, most quadruped robots can move quickly and steadily on both flat and undulating ground; however, natural environments are complex and changeable, so it is important for a quadruped robot to be able to jump over obstacles immediately. Inspired by the jumping movement of quadruped animals, we present aerial body posture adjustment laws and generate animal-like jumping trajectories for a quadruped robot. Then, the bionic reference trajectories are optimized to build a trajectory library of a variety of jumping motions based on the kinematic and dynamic constraints of the quadruped robot. The model predictive control (MPC) method is employed by the quadruped robot to track the optimized trajectory to achieve jumping behavior. The simulations show that the quadruped robot can jump over an obstacle of 40 cm in height. The effectiveness of the animal-like jump control method is verified.

## 1. Introduction

In nature, quadruped animals have the ability to handle discontinuous terrains with agility. For example, dogs can jump over hurdles. Although quadruped robots and quadruped animals are quite different in their driving modes and structures, their motion patterns and dynamic characteristics are similar. Both are driven by the ground reaction force (GRF) generated by four legs. Therefore, the movement characteristics of quadruped animals can inspire the control of quadruped robots. 

Due to the demand for motion performance, quadruped robots’ motion control has undergone a transition from quasi-static walking to highly dynamic walking. The demand for adaptation to complex environments presents challenges for both the stability and agility of quadruped robots. Highly dynamic jumping can deal with a variety of complex environments to improve quadruped robots’ agility [1,2,3,4,5]. Jumping behavior requires high speeds to be attained in a short time, which is associated with challenges related to physical constraints such as joint configuration and the friction cone. Traditional control methods fail to deal with this challenge. For example, the spring-loaded inverted pendulum (SLIP) model treats the animal jumping model as a spring-loaded inverted pendulum and decouples the jumping height, velocity, and Euler angle controls. However, the SLIP-based approach does not consider the friction cone. The optimization-based control method can effectively integrate constraints and targets. In this paper, trajectory optimization and model predictive control are used to control and generate the dynamic jumping behavior of a quadruped robot.

The quadruped robots that achieved the jumping motions include MIT Cheetah 3 [1] and Mini Cheetah [2]. Cheetah 3 planned and completed a 30-inch platform jump in a sagittal plane, and Mini Cheetah optimally generated and performed multiple jumps, such as jump platforms, barrel rolls, etc. The two robots solved optimization problems by following a pre-designed reference trajectory and used gradient optimization to generate a jumping motion that can be followed. The trajectory optimization approach proposed by ETH introduced the contact sequence into the optimization, utilizing the duration of the touchdown phase and realizing the traverse of a variety of complex terrains [6]. Based on the single rigid body model, MIT carried out whole-body trajectory optimization by taking the contact sequence and phase duration into account, thus realizing a high-altitude bidirectional rollback [7]. The Chinese University of Hong Kong used a heuristic optimization method without a reference trajectory to find a reasonable trajectory for jumping through various obstacles (windows and rectangles), but the accuracy of the landing position was insufficient [5]. Although these quadruped robots achieved the jumping behaviors, they made less use of biomimetic mechanisms. In fact, many institutions hoped to realize robot jumping behaviors by studying bionics. NASA imitated frog jumping and developed a froglike bionic jumping robot [8]. The FESTO company developed a bionic kangaroo robot [9], but their bionics mainly focused on body structures. The bionics of motion behaviors require studying the movement mechanisms of animals in great depth. Hildebrand studied cheetah movements and obtained the foot trajectory of cheetahs running at a gallop [10]. Stelian Coros captured motion data from dogs and realized various gait movements in a simulation environment [11]. The central pattern generator (CPG) control method was derived from the animal rhythmic motion control mechanism. CPG has advantages in control tasks, such as multi-freedom coordination and gait transition [12].

In this paper, we aimed to identify the potential jumping mechanism of quadruped animals and apply it to generate an animal-like reference trajectory, so that the motion behavior of the robot is more natural and is able to observe the motion mechanism of the long-term evolution of animals.

The main contributions of this paper are as follows:

- The reference trajectory of jumping is generated by integrating animal bionic information and the foot reaction forces of the robot. The variation in the body pitch angle of a dog was extracted from a motion video, then mapped to the robot’s body pitch angle. The robot’s foot reaction force was planned in the form of centroid momentum.
- The bionics-based trajectory was optimized based on kinematic and dynamic constraints. The jumping motion of a quadruped robot was realized using the model predictive control method in the simulation environment.

The rest of this paper is organized as follows. The second section introduces trajectory optimization based on bionics. The third section introduces the MPC trajectory controller. The fourth section details the implementation. The fifth section presents the results of the simulation. The sixth section is the conclusion.

## 2. Trajectory Optimization Based on Bionics

The body structure and movement patterns of quadruped animals in the natural world have always inspired the development of robots. Through the knowledge of bionics, quadruped robots of different structures have been developed, and control methods based on bionics have been presented [13]. In order to realize the jumping motion of a quadruped robot, we explored bionics to find the general mechanism whereby animals jump, then applied this mechanism to the control of a quadruped robot to achieve a better motion performance.

### 2.1. Jumping Process

**Figure 1:**
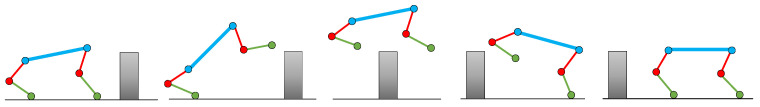
The process whereby a quadruped robot jumps over obstacles.

The ability to jump over obstacles and across ditches is achieved with the help of a jump controller. The jumping process is divided into four stages, as shown in Figure 1. The first stage is the four-legged standing stage, which is the preparation phase before the robot jumps. The second stage is the hind-leg standing stage, where the two front legs are pushed off the ground so that the robot body produces a large pitch angle while the two hind legs are flexed, and the robot is ready to jump into the aerial phase. The third stage is the aerial stage, in which the robot’s legs are contracted, and the whole body is in the air, subject only to the effect of gravity, with the explosive force in the supporting phase driven by inertia over obstacles or ditches. The fourth stage is the landing stage. The legs are extended for touchdown detection and as a landing buffer, and the quadruped robot detects the rotation speed of the body after the impact, performs a timely adjustment of the horizontal force of the foot to offset the extra rotational momentum, and adjusts the body posture to allow the robot to smoothly transition to its normal running gait. The whole jumping process is completed, and the quadruped robot smoothly passes over the obstacles and ditches.

**Figure 2:**
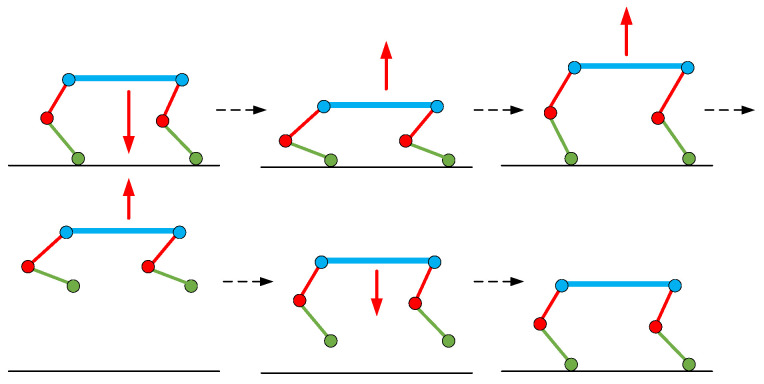
The vertical jump process of quadruped robots. The red arrow shows the tendency of the body to move in the vertical direction.

The process of vertical jumping is similar to that of jumping over obstacles. In the process of jumping, the robot uses four legs to exert force at the same time and does not change the pitch angle of the body. The pitch angle is set to zero. The vertical jump process is shown in Figure 2.

The movement patterns of quadruped animals can provide a reference for the behavior of quadruped robots. We incorporate the locomotion mechanism of quadrupedal animals into the trajectory optimization process as a pattern. Trajectory optimization, as a feed-forward input to model predictive control, can provide joint torques, the centroid’s position, and foot reference trajectories that satisfy the actual physical constraints as well as the possible ones.

Since the robot is only affected by the force of gravity in the air stage, it is difficult to change its posture in the air. Therefore, the position of the center of mass (COM) of the quadruped robot while landing depends only on the speed at the end of the jump. For that reason, the whole jump process can be seen as a motion process determined by the jump phase. Therefore, we focused on the phase when the quadruped takes off.

In this paper, we aimed to conduct optimization of the specified motion forms to generate the information that satisfies physical constraints and can be used for MPC. Consequently, reference motion trajectories should be specified before optimization, including the ground reaction, centroid trajectory, foot trajectory, etc.

#### 2.1.1. Ground Reaction Force during Takeoff

By considering the quadruped robot as a particle model whose motion during the take-off stage approximates the ballistic motion, the state of motion at the end of the take-off phase can be obtained, depending on the desired jump targets, namely distance and height. The velocity value of the center of mass achieved by the quadruped robot through GRF is as follows:

(1) $$△z_{des}=\frac{1}{2}g{t_{Flight}}^{2}\;v_{z}=gt_{Flight}$$

$$△x_{des}=v_{x}t_{Flight}$$

where $t_{Flight}$ is the duration of the airborne phase, $△z_{des}$ is the expected vertical displacement, $△x_{des}$ is the expected horizontal displacement, *g* is the gravitational acceleration, and $v_{x}$ and $v_{z}$ are the horizontal and vertical velocities, respectively.

Since the pose of the robot is not considered, we tend to assume that the ground reaction point is at the center of mass and then assign it to each foot through the contact sequence. If the form of the ground reaction is known, its specific expression function can be obtained through momentum conservation.

**Figure 3:**
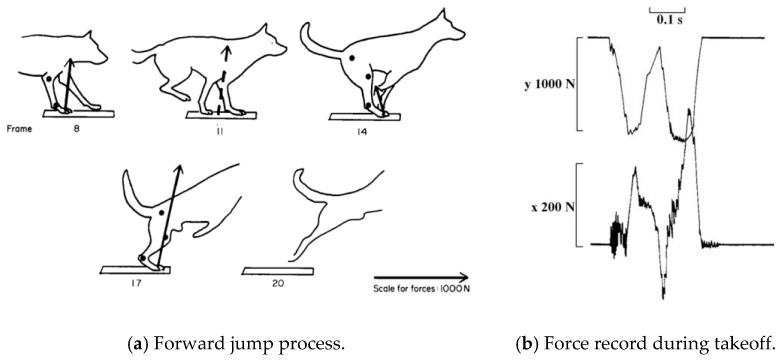
Research on the forward jump of an Alsatian dog; (**a**) tracings from the film of the long jump, (**b**) the force record during takeoff [14].

According to the tetrapod (Alsatian dog) studied by Alexander [14], the obstacle jumped forward, with a total length of 2.74 m and a height of 0.2 m. The jumping stage is shown in Figure 3a, which can be divided into the following phases: the front leg touching the ground, the back leg touching the ground, the front leg lifting off the ground, and the back leg lifting off the ground. In the last two processes, the pitch angle gradually increases. The ground reaction curve of the foot is shown in Figure 3b, where *y* represents the vertical direction, and *x* represents the horizontal direction. The maximum ground reaction of the foot is 1120 N in the y-direction and 230 N in the *x*-direction, and the former is about three times the weight of the dog (36 kg). The change in the force is quite drastic over a short time, and the trend of its change is approximately linear.

Therefore, we assume that the foot force changes approximately linearly when it makes contact with the ground, namely:

(2) f(t)=At+B

According to the momentum theorem and the initial state equilibrium, the reference ground reaction can be solved given the lasting time of the ground touching phase $t_{TO}$.

(3) mv=∫f(t)dt

The mapping between the robot’s force and motion state is generated by simplifying the single rigid body (SRB) model. The reference ground reaction force above can be mapped to the reference position information of the robot through the dynamic equation, and the reference pose information of the robot can be determined as follows.

#### 2.1.2. The Pitch Angle of the Body Changes during the Jump

**Figure 4:**
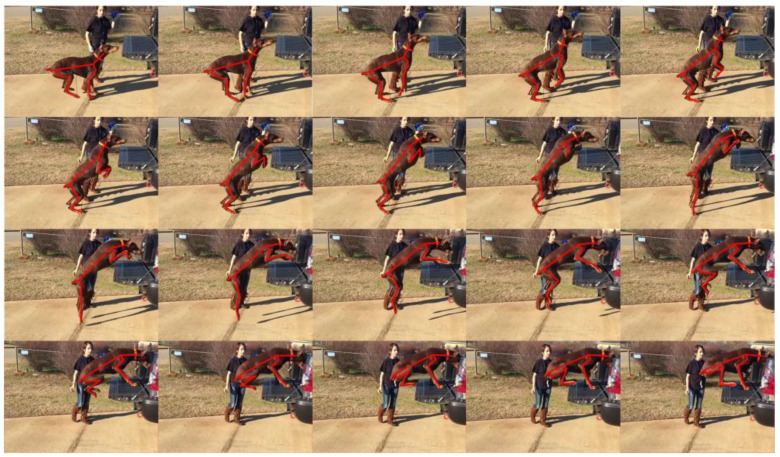
Frame capture of a dog jumping onto a platform to extract the position information of the trunk and legs.

**Figure 5:**
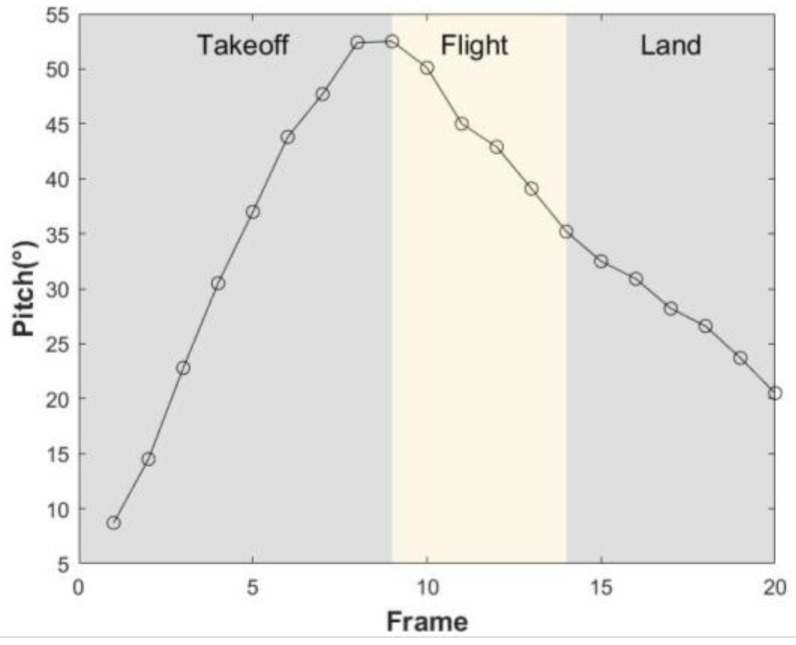
Changes in the pitch angle of the dog’s body.

The variation rule for the pitch angle in the jumping phase is obtained by processing the video of a quadruped jumping onto a platform using frame segmentation, as shown in Figure 4. Taking the horizontal direction as a reference, it was found that the pitch angle of the body increased from 8.5° to 52° after the front legs lifted off the ground, as shown in Figure 5.

We carried out trajectory tracking optimization on the specified motion form to generate information that satisfies the physical constraints and is convenient for the MPC to track. Therefore, the reference motion trajectory needs to be specified before optimization.

For forward jumping, we can ignore changes in the roll angle and yaw angle and only consider the pitch angle as approximate linear:

(4) φ=m t+n

where φ is the pitch angle of the robot body in the world frame, *t* is the time when the robot jumps and *m* and *n* are parameters that can be calculated from the changes in the pitch angle of the quadruped.

Considering that the initial pitch angle is 0 degrees, i.e., *n* = 0, the pitch angle at the end of takeoff is related to the obstacle height *h*. Assuming that the joint length between the foot and the hip joint of the foreleg is *L* when the hind leg is fully extended off the ground, the pitch angle during takeoff is defined as:

(5) $$\varphi_{TO}=\sin^{-1}(\frac{h}{L})$$

### 2.2. Trajectory Planning and Optimization

#### 2.2.1. Single Rigid Body Model

Unlike robotic arms, which control the position and pose of the end claw to reach an object, legged robots focus on the movement of the body. The body itself is movable under the world system, so a legged robot is a moving object with a floating base. The quadruped robot interacts with the world and generates motion only by the ground reaction force of the feet. When modeling the dynamics of a quadruped robot, it can be simplified as a single rigid body model, which is only affected by the ground reaction force and gravity of the sole of the foot. 

**Figure 6:**
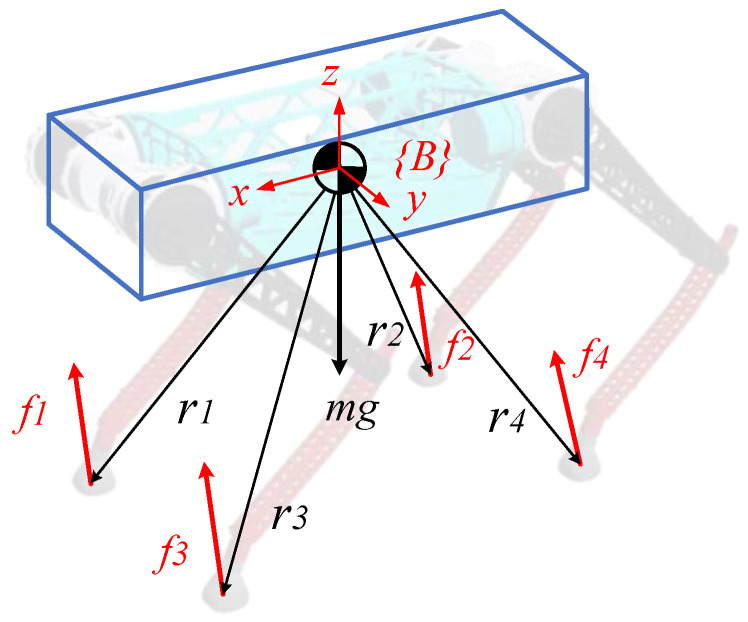
Single rigid body model of a quadruped robot.

The robot is simplified into a single rigid body model, as shown in Figure 6. The dynamic equation for the single rigid body model is obtained in terms of Newton’s second law. According to the angular momentum theorem, the rotation equation of the single rigid body model is obtained. The dynamics of a rigid body in world coordinates are given as:

(6) $${\ddot{p}}_{\mathrm{com}\;}=\frac{\sum_{i=1}^{4}f_{i}}{m}+g$$

ddt(IP·ωOB)=∑i=14ri×fi+03×1×(m·g)

where ${\ddot{p}}_{\mathrm{com}\;}$ is the robot’s acceleration, m is the robot’s mass, g is the acceleration of gravity, IP is the robot’s inertia tensor under the directional local system, $\omega_{OB}$ is the angular velocity of the robot’s machine system relative to the world system, r is the position vector of the foot relative to the center of mass in world coordinate, and f is the ground reaction force on the robot. The coordinates of frame *P* are fixed at the body’s center of mass in the same direction as the world frame. The symbol does not have a left superscript, meaning that it is the default under the world frame.

The state of the SRB model of the robot is given by:

(7) $$x={\left[p^{T}\theta^{T}{\dot{p}}^{T}\omega^{T}\right]}^{T}$$

#### 2.2.2. Trajectory Optimization Method

In trajectory optimization, the information in (7), combined with bionics, is integrated. The cost function of optimization is mainly to track the reference information, as follows:

(8) $$\begin{array}{l} \min\;\;\;\sum_{k=1}^{N-1}[(x_{k}-x_{refk}{)}^{T}Q_{x}(x_{k}-x_{refk})+{\left(u_{k}-u_{refk}\right)}^{T}Q_{u}(u_{k}-u_{refk})]\\ +{\left(x_{N}-x_{refN}\right)}^{T}Q_{N}(x_{N}-x_{refN})\;\;\;\; \end{array}$$

where the subscript *ref* represents the reference trajectory, the subscript *N* represents the state at the last moment, and *k* represents the physical quantity at the *k* moment. x is the corresponding system state in the single rigid body model and u represents the robot control input, including the robot foot position *c* and the ground reaction force *f*:

(9) $$u={\left[c_{1}{}^{T},f_{1}{}^{T}\ldots \;\ldots c_{4}{}^{T},f_{4}{}^{T}\right]}^{T}$$

In order to ensure that the solution to the optimization problem conforms to the actual physical situation as much as possible, constraints are imposed on the optimization problem, which are expressed as follows:

The mapping relationship between the robot state quantity and the control quantity satisfies the simplified constraints of discrete single rigid body dynamics. The quantities of states at different moments can then be represented by the discrete SRB model: the subscript *k* represents moment *k*. SRB (6) constraints are integrated into trajectory optimization via Euler integration of the robot’s state between time steps:

(10) $$x(k+1)-x(k)=\dot{x}(k)\Delta t$$

where $\dot{x}$ is the set vector of state derivatives which can be used to derive the state at the next moment:

(11) x˙=ddt[pθp˙ω]=[p˙B(θ)ω1mf−gI−1B(RTBOτ−ωB×IBωB)]

where $B\in R^{3\times 3}$ is an orientation-dependent matrix that converts angular velocity to Euler angle rates, RBO is the rotation matrix from the body frame *B* to the world frame *O*, θ is the Euler angle representing the orientation of the SRB, and ω× is defined as the skew-symmetric matrix.

The detailed form of Formula (10) is as follows:

(12) p(k+1)−p(k)=p˙(k)Δtθ(k+1)−θ(k)=B(θk)−1RBO(θk)Δtp˙(k+1)−p˙(k)=p¨(k)Δtω(k+1)−ω(k)=ω˙(k)Δt

The positions of the feet of the robot and the position of the centroid of the torso are mapped kinematically through the joint angle, where $c_{i,}{}_{k}$ is the position of the foot of the leg *i* of the robot at moment *k*, and $\gamma (q_{k})$ is the mapping function for determining the position of the foot through the joint position:

(13) $$c_{i,}{}_{k}-\gamma (q_{k})=0$$

When the robot’s feet are in contact with the ground, it is expected that the feet will not slip on the ground, in line with the friction cone constraint. Moreover, the ground reaction can only push the robot, but not pull it:

$$c_{touch}-\mathrm{map}(z)=0\;c_{k+1}-c_{k}=0$$

(14) $$-\mu f_{z,k}\leq f_{x,k}\leq \mu f_{z,k}$$

$$-\mu f_{z,k}\leq f_{y,k}\leq \mu f_{z,k}\;\;-\;f_{z}\leq 0$$

where μ is the Coulomb friction factor, $c_{touch}$ represents the position of the foot that is in contact with the ground, and map(*z*) is the height function of the terrain.

Considering that the optimization results should be applied to the actual robot, the physical configuration of the robot is reflected in the constraints, where $\tau_{\max}$ is the maximum torque of the robot joint,$J_{k}$ is the contact Jacobian matrix, and q is the joint angle of the robot:

(15) $$\;\left|J_{k}{}^{T}f_{k}\right|-\tau_{\max}\leq 0$$

(16) $$\left|q_{k}\right|\leq q_{\max}$$

## 3. Model Predictive Control

The model predictive controller was designed for the improved performance of the quadruped robot. Unlike the instantaneous SLIP [15], VMC [16,17], and WBC [18] controllers, the MPC controller can predict the future state of the robot based on the variables and system model [19]. The quadruped robot is an intermittent underactuated system in the process of walking or running.

Predictive control is particularly necessary for jump motions with long aerial phases. In the takeoff phase, the quadruped robot is only affected by gravity, and the motion of the leg joints hardly changes the trajectory of the center of mass. Therefore, it is necessary to prepare for the takeoff phase in the takeoff phase so that the robot can predict the state when it is in the air to reduce the trajectory tracking error as much as possible. In other words, the control parameters are calculated according to the feedback and expected values in the current control cycle in the takeoff phase. It is not only necessary to achieve this aim so that the robot can track the expected position in the current control cycle but also determine the number of control parameters in the future.

### 3.1. Equation of State and Discretization

The MPC uses the equation of state to model the robot. The equation of state is a first-order differential system composed of the state variables of the system, which are derived from the physical mechanism of the system. However, the actual single rigid body dynamic equations are nonlinear complex equations, including the angular momentum theorem and the angular velocity equation. Therefore, the equations need to be simplified and linearized.

In the model predictive controller, the states of the system x are the same as those defined in the trajectory planning, and the control inputs of the system u are expressed as:

(17) $$u={\left[f_{1}^{T}f_{2}^{T}f_{3}^{T}f_{4}^{T}\right]}^{T}$$

The linear equations of state in continuous time can be constructed from the dynamic equations of the single rigid body model of the robot:

(18) $$\dot{x}(t)=A_{c}(\psi )x(t)+B_{c}\left(r_{1},r_{2},r_{3},r_{4},\psi \right)u(t)$$

where $A_{c}$ and $B_{c}$ are expressed as:

(19) Ac=[03×303×3RzT(ψ)03×3003×303×303×3I3⋮03×303×303×303×3003×303×303×303×310⋯0…0],Bc=[03×303×303×303×303×303×303×303×3IP−1⋅r1×IP−1⋅r2×IP−1⋅r3×IP−1⋅r4×I3/mI3/mI3/mI3/m01×301×301×301×3]

In the equation of state, matrix $A_{c}$ is the relation of the internal states of the system, which is called the system matrix, and matrix $B_{c}$ is the effect of the input on the state, which is called the input matrix or control matrix.

The equation of state in continuous time is discretized as:

(20) $$\dot{x}=\frac{x(k+1)-x(k)}{\Delta T}=Ax(k)+Bu(k)$$

where ΔT is the period of model predictive control.

The equation of state for the next state can be solved via iterations:

(21) $$x(k+1)=(I+\Delta T\cdot A)x(k)+\Delta T\cdot Bu(k)=A_{k}x(k)+B_{k}u(k)$$

By combining all the discrete equations of state in the prediction horizon, the prediction equation of model predictive control can be obtained:

(22) $$X=A_{qp}x_{0}+B_{qp}U$$

where $x_{0}$ is the current state at the moment, obtained from the state estimator. U is the vector of all control inputs during the prediction horizons. X is the vector of all states during the prediction horizon. $A_{qp}$ is state coefficient matrix, and $B_{qp}$ is control coefficient matrix. The values of each item are:

(23) $$X={\left[x_{1}^{T}x_{2}^{T}\cdots x_{h}^{T}\right]}^{T},U={\left[u_{1}^{T}u_{2}^{T}\cdots u_{h-1}^{T}\right]}^{T}\;A_{qp}=\left[\begin{array}{c} A_{0}\\ A_{1}A_{0}\\ A_{2}A_{1}A_{0}\\ \vdots \\ \prod_{i=h-1}^{0}A_{i} \end{array}\right],B_{qp}=\left[\begin{array}{cccc} B_{0} & 0 & \cdots & 0\\ A_{1}B_{0} & B_{1} & \cdots & 0\\ \vdots & \vdots & \ddots & 0\\ \left(\prod_{j=h-1}^{1}A_{j}\right)B_{0} & \left(\prod_{j=h-1}^{2}A_{j}\right)B_{1} & \cdots & B_{h-1} \end{array}\right]$$

### 3.2. Optimization Problem Construction and Solution

Based on the prediction equation, the weighted least squares optimization method is used to solve the control inputs, that is, the GRF of the stand phase. The optimization problem is:

(24) $$\begin{array}{c} \min_{U}J(U)={\left(X-X_{ref}\right)}^{T}Q(X-X_{ref})+U^{T}RU\\ s.t.\;f_{i}=0_{3\times 1},\;\;\;\forall s_{i}S_{\Phi }=0\\ {\underline{c}}_{i}⩽C_{i}\cdot f_{i}⩽\bar{c} \end{array}\;$$

where J(U) is the objective function of the optimization problem. $X_{ref}$ refers to the states that the robot is expected to achieve in the prediction horizon. C is the coefficient matrix of friction constraints. The first constraint equation indicates that the ground reaction force is directly assigned to zero when the leg is in the swing phase.

The optimization objective function of MPC is in the quadratic convex optimization form. The ***Q*** matrix is the state-weighted matrix, and the ***R*** matrix is the control-weighted matrix. In engineering practice, ***Q*** and ***R*** are symmetric matrices. We chose ***Q*** and ***R*** as diagonal matrices. The smaller the error in the expected state component, the larger its weighting coefficient should be. If the error is weighed differently at different stages in the dynamic process, the corresponding weighting coefficient should be a time-varying coefficient. The latter term of the objective function represents the constraints on the control input. U is as small as possible. The objective function *J* essentially uses a small control input to maintain a small error, so as to achieve the optimal synthesis of energy and the tracking error.

The optimization equation can be written in the form of quadratic programming:

(25) $$\begin{array}{c} \min_{U}J(U)=\frac{1}{2}U^{T}HU+U^{T}g\\ s.t.\;\underline{c}⩽CU⩽\bar{c} \end{array}\;$$

The control matrix is calculated using the general quadratic programming solution library. Then, all GRFs in the prediction horizons are obtained.

## 4. Controller Implementation

The proposed bionics-based jump-control method is applied to the quadruped robot BQR3. In this section, the relevant parameters and control block diagram of the robot are introduced.

### 4.1. Robot Platform

**Figure 7:**
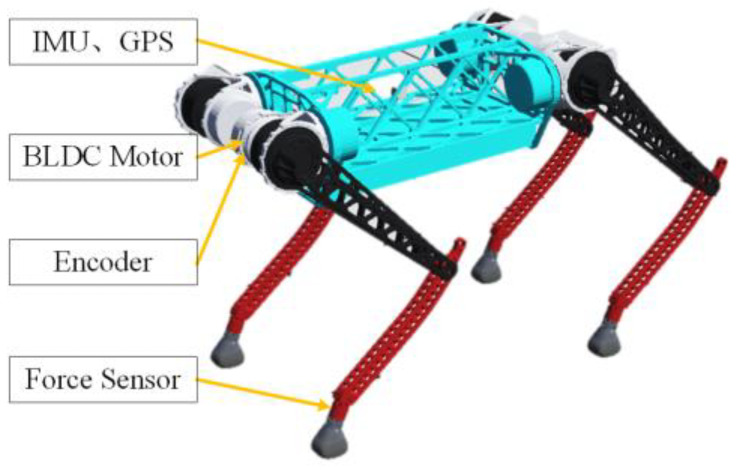
Quadruped Robot BQR3.

The simulation object we used is the BQR3 quadruped robot, as shown in Figure 7. BQR3 has 12 degrees of freedom. The robot is driven by a BLDC motor. The position and velocity of the joints are obtained using encoders. The angular velocity and linear acceleration of the robot are obtained using an IMU, and the GRFs are obtained using six-dimensional force sensors mounted on the feet. The physical parameters are shown in Table 1.

### 4.2. Control Framework

**Figure 8:**
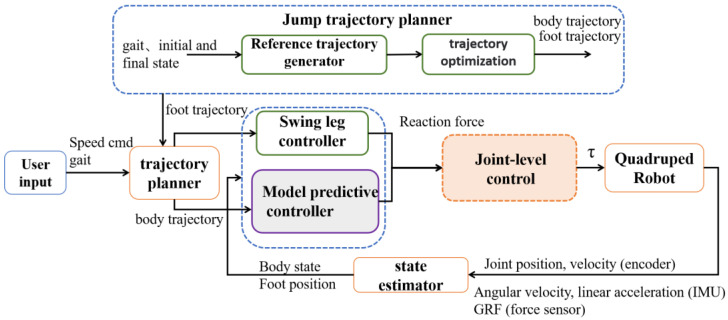
Control framework. The MPC runs at 100 Hz. The joint controller runs at 1 kHz.

The overall control block diagram is shown in Figure 8. Users input control instructions and send them to the robot to make the robot execute corresponding actions. According to the input instruction, the trajectory planner computes the relevant trajectories and sends them to the bottom tracking controller, namely the swing leg controller and the model prediction controller. The tracking controller calculates the required foot force and then maps it to the joint torques through the joint controllers; it then sends the joint torques to the robot, and the robot performs the corresponding actions. The state estimator feeds back the robot’s current state values in real-time.

### 4.3. Leg Aerial Phase Control

When the leg is in a swing phase, the swing leg controller calculates and tracks the desired footpath, using a PD feedback control to minimize the errors in real-time.

(26) $$f_{swing}=K_{p}(p_{ref}-p)+K_{d}(v_{ref}-v)$$

(27) $$\tau_{swing}=J^{T}f$$

where $p_{ref}$ and p are the desired and actual foot positions; $v_{ref}$ and v are the desired and actual foot velocities; and $K_{p}$ and $K_{d}$ are the PD coefficients.

### 4.4. Self-Stabilizing Balance Control of the Landing

The plantar force sensor is used for the landing of the robot. During landing, when the GRF measured by the force sensor is greater than the minimum threshold, that is, $f_{i}>20N$, it is judged that the foot has touched the ground. When the number of contact legs is greater than two, the quadruped robot state machine switches from the aerial phase to the four-legged standing phase. In addition, in order to prevent unstable standing, after the planned jumping time is over, the robot immediately switches to a trot gait to maintain its dynamic balance. The robot can attain balance quickly and stably after the significant landing impact.

## 5. Experiment Results

### 5.1. Trajectory Optimization Results

**Figure 9:**
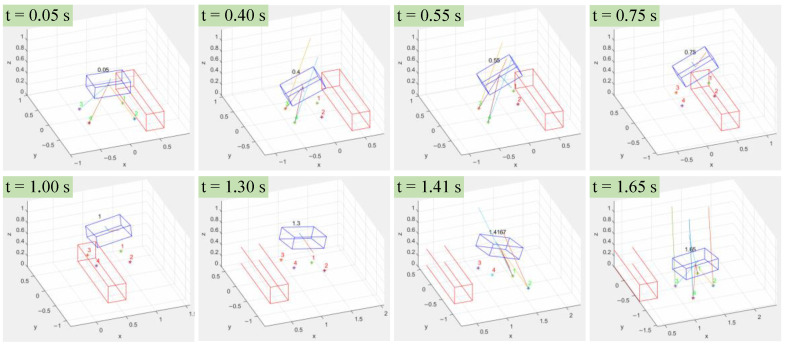
Snapshots of the quadruped robot jumping motion.

The simulation objectives of the quadruped robot jumps are a 1.5 m long jump, a 1m-body-height high jump, and successfully crossing a 40 cm high obstacle. For the trajectory optimization of the jumping process, the nonlinear optimization solver library CasADi [20] was used to solve the trajectory parameters in MATLAB (R2021a). The jump trajectories were obtained by optimizing the robot’s offline jump motion. The time of each stage in the forward hopping process is planned in advance, as shown in Table 2:

The offline optimization process uses AMD R7 4800 H CPU, and the total optimization time is 48 s. The results of the numerical simulation are shown in Figure 9; the time of each stage of the jumping process is consistent with the planned ones.

**Figure 10:**
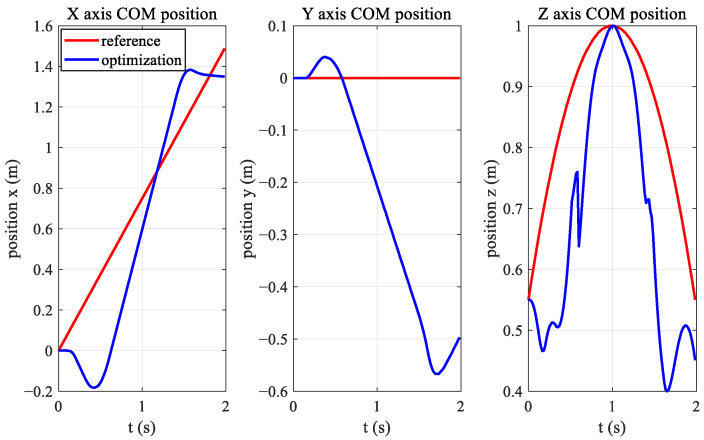
Position curve of the quadruped robot’s center of mass. The red curve is the value of the centroid reference position, and the blue curve is the centroid track after trajectory optimization.

Figure 10 shows the change in the centroid position during the jumping process. The red curve is the value of the reference trajectory, and the blue curve is the optimized trajectory. The long jump in the *x*-direction reached 1.4 m, 0.1 m less than the target of 1.5 m. The vertical jump reached the desired height of 1.0 m or body height. 

**Figure 11:**
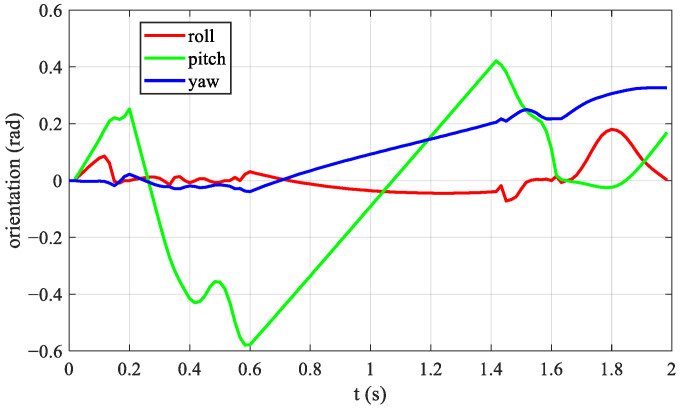
Quadruped robot’s RPY angle. The red curve is the change in the roll angle, the green curve is the change in the pitch angle, and the blue curve is the change in the yaw angle.

However, due to nonlinear optimization, the foot positions of the two hind legs are not parallel during the jumping process, and the foot force of the two hind legs are different. This affects the yaw angle of the body when the robot jumps forward. The changes in body posture during jumping are shown in Figure 11, where the red curve is the change in the roll angle, the green curve is the change in the pitch angle, and the blue curve is the change in the yaw angle. The pitch angle varied significantly, reaching −0.6 rad during the hind-leg support phase, which applies the law of variation in bionic studies.

**Figure 12:**
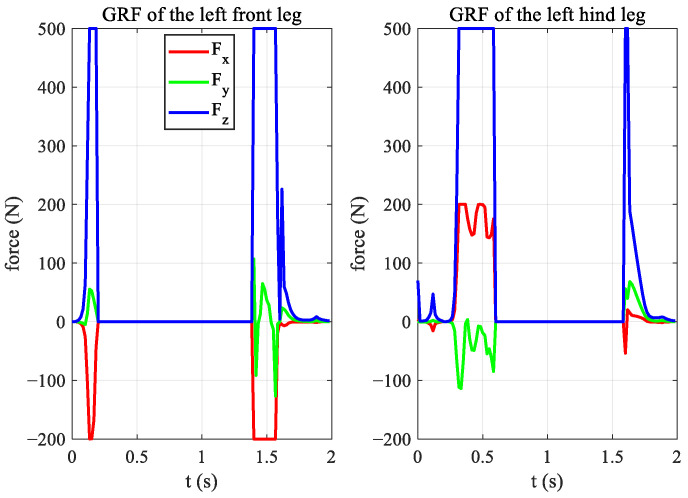
GRF of the forward jumping of the quadruped robot, BQR3.

The variation in the GRF during the jump is shown in Figure 12. The left figure shows the force data of the left front leg, and the right figure shows the data of the left hind leg of the robot. The red curve is the change in the *x*-direction, the green curve is the change in the *y*-direction, and the blue curve is the change in the *z*-direction. It can be seen that the forward jump mainly requires upward and forward ground forces, with the upward force being the largest.

### 5.2. Simulations of Jumps

**Figure 13:**
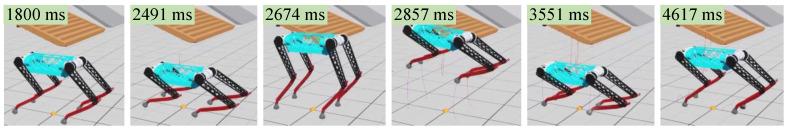
Snapshots of BQR3’s jumping motion.

The MPC uses a 15-step prediction horizon and a time step of 0.01s and is solved using qpOASES [21] at 1000Hz online. The dynamic simulations were carried out using the Webots software. The target of the vertical jump is 1 m in height with four legs exerting force at the same time. The dynamic simulations are shown in Figure 13.

**Figure 14:**
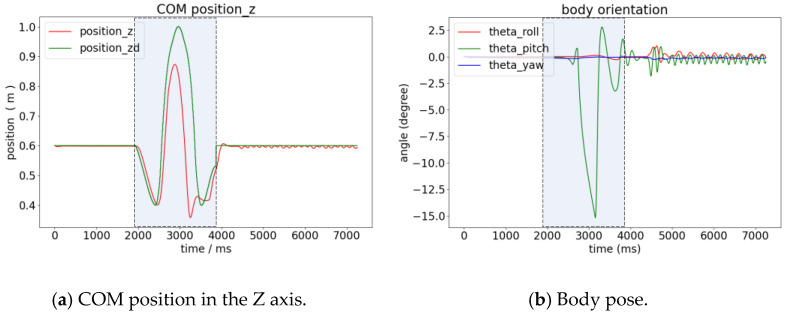
The position and pose changes in the jumping process; (**a**) shows the desired position (green curve) and actual position (red curve) changes in the vertical direction of the center of mass; (**b**) is the change in the value of the Euler angle of the robot. The gray zone shows the process from the takeoff phase to the landing phase.

The curves of the robot body in the vertical jump are shown in Figure 14. The gray zone is the jumping process. The time before the gray part is the preparation stage, and the time after the gray part is the stage when the balance is recovered after the landing when the trot begins. The jump height is close to 0.9 m, 0.1 m lower than the desired value. The pitch angle of the robot body has a fluctuation of 15°.

**Figure 15:**
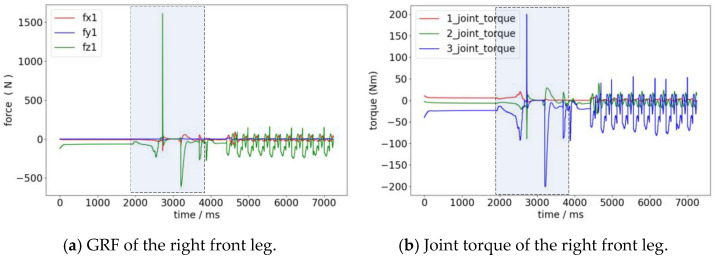
Changes in the ground reaction force and joint torque of the right front leg during jumping. The gray zone shows the process from the takeoff phase to the landing phase.

The ground reaction forces and joint torques of the right front leg are shown in Figure 15. When the four legs take off at the same time for a vertical jump, the maximum forces need to be exerted in the vertical direction, and the knee joints need to provide the maximum torques.

## 6. Conclusions

In this paper, we focus on the jumping motion of a quadrupedal robot. We established the laws of body trajectories and ground reaction forces during the jumping process using bionic data and further obtained the bio-inspired reference trajectories for quadrupedal robot jumping. The bionic reference trajectory was optimized to generate the executable trajectory of a jumping motion based on kinematic and dynamic constraints. The model predictive control method was used to track the optimized trajectory. The jump control algorithm was applied to the quadruped robot, BQR3. The vertical jump and forward jump were realized in the dynamic simulation environment. Generating a highly dynamic and agile motion like a jump is a challenging task. Bioinspired laws are useful tools for solving this difficulty and improving robots’ performances. The approach presented in this paper has the potential to be applied to real robots in the future. 

## Figures and Tables

**Table 1:** The parameters of the BQR3 Robot.

Parameter	Symbol	Value	Units
Mass	m	40	kg
Body inertia	$\left[I_{xx},I_{yy},I_{zz}\right]$	[0.195, 0.58, 0.615]	$\mathrm{kg}\cdot m^{2}$
Length of the body	$\left[l_{body},w_{body},h_{body}\right]$	[600, 300, 200]	mm
Length of the leg	$\left[l_{0},l_{1},l_{2}\right]$	[80, 360, 360]	mm

**Table 2:** Time planning of each stage in the forward jump process.

Phase	Time/s
Four-legged standing stage	0.2
Hind-leg standing stage	0.4
Aerial stage	0.8
Front-leg touchdown stage	0.2
Four-leg touchdown phase	0.4

## Data Availability

The data used to support the findings of this study are available from the corresponding author upon request.
